# The Effect of pH on Atenolol/Nanofiltration Membranes Affinity

**DOI:** 10.3390/membranes11090689

**Published:** 2021-09-06

**Authors:** Elisa Veridiani Soares, Alexandre Giacobbo, Marco Antônio Siqueira Rodrigues, Maria Norberta de Pinho, Andréa Moura Bernardes

**Affiliations:** 1 Post-Graduation Program in Mining, Metallurgical and Materials Engineering (PPGE3M), Federal University of Rio Grande do Sul (UFRGS), Av. Bento Gonçalves, n. 9500, Agronomia-Porto Alegre-RS, CEP 91509-900, Brazil; elisavsoares@gmail.com (E.V.S.); amb@ufrgs.br (A.M.B.); 2 Centre of Physics and Engineering of Advanced Materials (CeFEMA), Instituto Superior Técnico, University of Lisbon, Av. Rovisco Pais, n. 1, 1049-001 Lisbon, Portugal; marianpinho@tecnico.ulisboa.pt; 3 Post-Graduation Program in Materials Technology and Industrial Processes, Pure Sciences and Technology Institute, Feevale University, Rodovia RS-239, n. 2755, Vila Nova-Novo Hamburgo-RS, CEP 93525-075, Brazil; marcoR@feevale.br; 4 Chemical Engineering Department, Instituto Superior Técnico, University of Lisbon, Av. Rovisco Pais, n. 1, 1049-001 Lisbon, Portugal

**Keywords:** atenolol, pharmaceutical compound, wastewater treatment, nanofiltration, membrane separation, solution-diffusion model, membrane-solute interaction

## Abstract

Nanofiltration has been shown to be effective in removing pharmaceutical compounds from water and wastewater, so different mechanisms can influence treatment performance. In the present work, we carried out a case study evaluating the performance of two nanofiltration membranes in the removal of Atenolol (ATN)—a pharmaceutical compound widely used for the treatment of arterial hypertension—under different conditions such as operating pressure, ATN concentration, and solution pH. By determining the *B* parameter, which quantifies the solute/membrane affinity, we verified that the solution pH influenced the performance of the membranes, promoting attraction or repulsion between the ATN and the membranes. At pH 2.5, both membranes and ATN were positively charged, causing electrostatic repulsion, showing lower values of the *B* parameter and, consequently, higher ATN rejections. At such a pH, the mean ATN rejection for the loose membrane (NF270) was 82%, while for the tight membrane (NF90) it was 88%. On the other hand, at 12 bar pressure, the NF70 membrane (5.1 × 10 ^−5^ m s^−1^) presented mean permeate fluxes about 2.8 times greater than the NF90 membrane (1.8 × 10^−5^ m s^−1^), indicating that NF270 is the most suitable membrane for this application.

## 1. Introduction

Over the last three decades, the occurrence of pharmaceutical compounds in the environment, as well as the different solutions aimed at removing these compounds from wastewater, thus preventing them from reaching the environment, have been the central theme of numerous scientific articles [1,2,3,4]. Among these solutions are treatments by advanced oxidative processes [5,6,7], membrane filtration technologies [8,9,10], biological processes [11,12,13], and combined processes [14,15].

Considering the current available alternatives, nanofiltration (NF) has stood out, as it has shown high efficiency in the removal of pharmaceutical compounds such as anti-inflammatories [16], antibiotics [9], and beta-blockers [17,18]. In the latter group, Atenolol (ATN)—a beta-blocker widely used for the treatment of cardiovascular diseases such as hypertension—is one of the most consumed drugs in the world, since about 30% of the world population suffers from hypertension problems [19]. This pharmaceutical compound is only partially absorbed by the human body—50–60% of an oral dose is absorbed by the gastrointestinal system and 40–50% is eliminated (unchanged) mainly via urine, while the remainder is excreted via feces [20]. As a result, the drug not absorbed by the patient’s body is released into the sewage collection network and enters the sewage treatment plants, which, in turn, are often inefficient in removing these compounds, and therefore reach the environment.

According to some studies, once ATN reaches the environment, it can cause adverse effects on the organisms living there. By carrying out toxicity tests, Bittner et al. [21] observed that up to 64% of zebrafish embryos exposed to ATN at concentrations below 10 mM did not exhibit an inflated swim bladder. In another study, using *D. Magna* as the test organism, Ji et al. [22] found that approximately 70% of individuals lost mobility when exposed to 40 μM of ATN for 48 hours. Therefore, considering the harmful toxicological effects caused by ATN on aquatic organisms, it is of paramount importance to remove it as well as other pharmaceutical compounds from polluting sources, such as domestic sewage and wastewater from hospitals, pharmaceutical industries, and medical and veterinary clinics, to prevent them from reaching the environment.

In fact, nanofiltration has been shown to be effective in removing Atenolol from wastewater [17,23]. However, there are several factors that affect its performance, and it is important to highlight that this membrane technology, in addition to being governed by physical mechanisms, it is also influenced by electrostatic interactions that, for example, can be caused and/or altered by varying the pH of the incoming wastewater [24]. Furthermore, solute/membrane interactions can also be described by the solution-diffusion model, where a *B* parameter is used to quantify the affinity of a given solute for the membrane [25]. In this context, the aim of this study is to evaluate the influence of pH on the removal of the Atenolol by nanofiltration process, as well as its influence on the affinity of the solute for the membrane through the determination of the *B* parameter.

## 2. Theory

**Figure 1:**
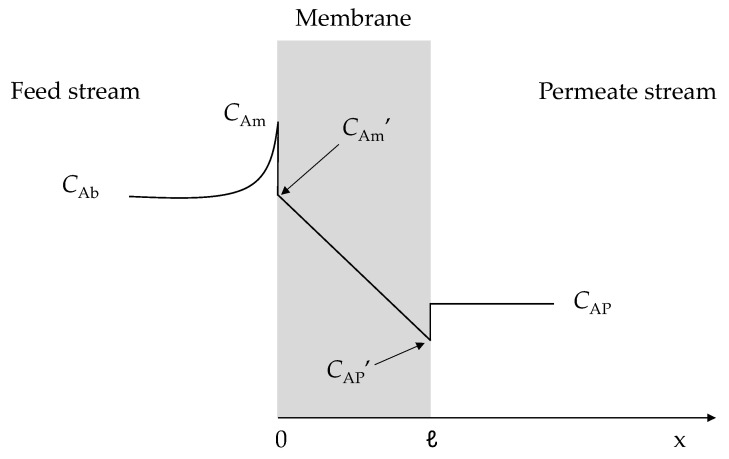
Scheme of the mass transport for a given solute A in an NF membrane by the solution-diffusion model. *C*_Am_’ and *C*_AP_’ are the concentrations of solute A inside the membrane on the feed and permeate sides, respectively. Adapted from [25].

The transport of a chemical species across a membrane is determined by the interactions between that species and the membrane material and by the difference in the chemical potential of that species between the two sides of the membrane. According to the solution-diffusion model, mass transport in NF membranes is based on a mechanism of sorption at the feed/membrane interface, diffusion across the membrane, and desorption at the membrane/permeate interface [26]. Therefore, as represented in Figure 1, the diffusive flux of a solute A (*J*_A_) through an NF membrane can be described by Fick’s law as follows [25]:

(1) $$J_{A}=-D_{\mathrm{Am}}\frac{\partial C_{A}}{\partial x}$$

where *D*_Am_ is the solute diffusivity inside the membrane, *C*_A_ is the solute concentration, and *x* is the spatial coordinate through the membrane.

The relationship between solute concentrations in fluid phases adjacent to the membrane (feed and permeate sides) and solute concentrations inside the membrane close to these phases is given by the partition coefficient (*Ф = C*_A_’/*C*_A_) [25]. 

By integrating Equation (1) along the membrane thickness and considering the boundary conditions, *x* = 0 → *C*_Am_’= *ФC*_Am_ and *x* = *ℓ* → *C*_AP_’ = *ФC*_AP_, yields:

(2) $$J_{A}=\frac{D_{\mathrm{Am}}\Phi }{\ell }\left(C_{\mathrm{Am}}-C_{\mathrm{AP}}\right)=B\left(C_{\mathrm{Am}}-C_{\mathrm{AP}}\right)$$

where *ℓ* is the membrane thickness, *C*_Am_ and *C*_AP_ are the concentrations of solute A at the membrane surface and in the permeate, respectively, and *B* is a parameter characteristic for a given membrane/solute system, so that *B* = *D*_Am_*Ф*/*ℓ*.

In steady-state conditions, the flux of solute A across the membrane can also be represented as:

(3) $$J_{A}=J_{P}C_{\mathrm{AP}}$$

where *J*_P_ is the permeate flux.

Combining Equations (2) and (3) and using the definition of intrinsic rejection for a given solute A ($f_{A}^{’}$. ), results in:


(4) $$f_{A}^{’}=\frac{J_{P}}{J_{P}+B}$$

Indeed, the selectivity of a membrane for a given solute A is usually measured by the intrinsic rejection coefficient,

(5) $$f_{A}^{’}=\frac{C_{\mathrm{Am}}-C_{\mathrm{AP}}}{C_{\mathrm{Am}}}$$

or by the apparent rejection coefficient (*f*_A_),

(6) $$f_{A}=\frac{C_{\mathrm{Ab}}-C_{\mathrm{AP}}}{C_{\mathrm{Ab}}}$$

where *C*_Ab_ is the solute concentration in the bulk feed solution. So, combining Equations (5) and (6), these rejection coefficients are related as

(7) $$f_{A}=\frac{f_{A}^{’}}{f_{A}^{’}+\left(1-f_{A}^{’}\right)\;\exp\left(\frac{J_{P}}{k}\right)}$$

and for the use of the pressure variation method [27], Equation (7) is rearranged as


(8) $$\ln\left(\frac{1-f_{A}}{f_{A}}\right)=\ln\left(\frac{1-f_{A}^{’}}{f_{A}^{’}}\right)+\frac{1}{k}\times J_{P}$$

Therefore, the linear regression of the experimental results of $\ln\left(\frac{1-f_{A}}{f_{A}}\right)$. vs. $J_{P}$. generates a straight line of slope 1/*k*, in which *k* is the mass transfer coefficient, and the intercept at the origin, $\ln\left(\frac{1-f_{A}^{’}}{f_{A}^{’}}\right)$, makes it possible to determine $f_{A}^{’}$. 

Then, after determining $f_{A}^{’}$ and rearranging Equation (4), the *B* parameter can be obtained:

(9) $$B=\frac{J_{P}\left(1-f_{A}^{’}\right)}{f_{A}^{’}}$$

## 3. Materials and Methods

Experimental runs were conducted using two NF membranes, NF90 and NF270, from Dow–FilmTec (Edina, MN, USA), in laboratory flat-cell units with a membrane surface area of 14.5 cm^2^, which was thoroughly described in previous studies [28,29].

The membranes used in this study had already been characterized and used in a previous work, where pure water permeabilities of 10.3 and 19.7 kg h^−1^ m^−2^ bar^−1^ and sodium chloride rejections of 90% and 49% were achieved for NF90 and NF270 membranes, respectively [17]. In fact, NF90 and NF270 are characterized as tight and loose NF membranes with a molecular weight cut-off (MWCO) of 200 Da and 400 Da and a pore radius of 0.34 nm and 0.42 nm, respectively [8,24,30].

Feed solutions containing 8 or 16 mg L^−1^ of ATN, representing concentrations usually found in wastewater from the pharmaceutical industry, were prepared in deionized water (conductivity less than 5 μS cm^−1^) at three different pH conditions: 2.5, 7.0, and 10.5. ATN with 99% purity purchased from a compounding pharmacy was used to prepare the feed solutions and the pH was adjusted with 0.1 M sulfuric acid or sodium hydroxide solutions. The main characteristics of ATN are reported in Table 1.

The NF experimental runs were carried out in batch mode, where the retentate and the permeate streams were recycled to the feed tank to evaluate the behavior of the permeate fluxes, *B* parameter, and solute rejection coefficients at 0.96 m s^−1^ (feed circulating velocity), under different operating pressures (6–12 bar), pH conditions (2.5, 7.0 or 10.5), and feed solution concentrations (8 or 16 mg L^−1^ ATN). Based on previous studies [17,33], the stabilization time in each experimental run was set as 30 min, after which feed and permeate samples were taken for chemical analysis. The ATN concentration was measured by a spectrophotometric method [34], whose calibration curve—ATN (mg L^−1^) = 23.167 absorbance (R^2^ = 0.999)—was obtained by measuring the absorbance at 226 nm (wavelength of maximum absorbance of ATN) in a spectrophotometer T80+ UV-Vis (PG Instruments, UK). All experiments and analyses were carried out at least in duplicate. The membranes were carefully cleaned between each run with deionized water or a pH 9.0 solution until the pure water flux reached at least 90% of the initial value.

## 4. Results and Discussion

**Figure 2:**
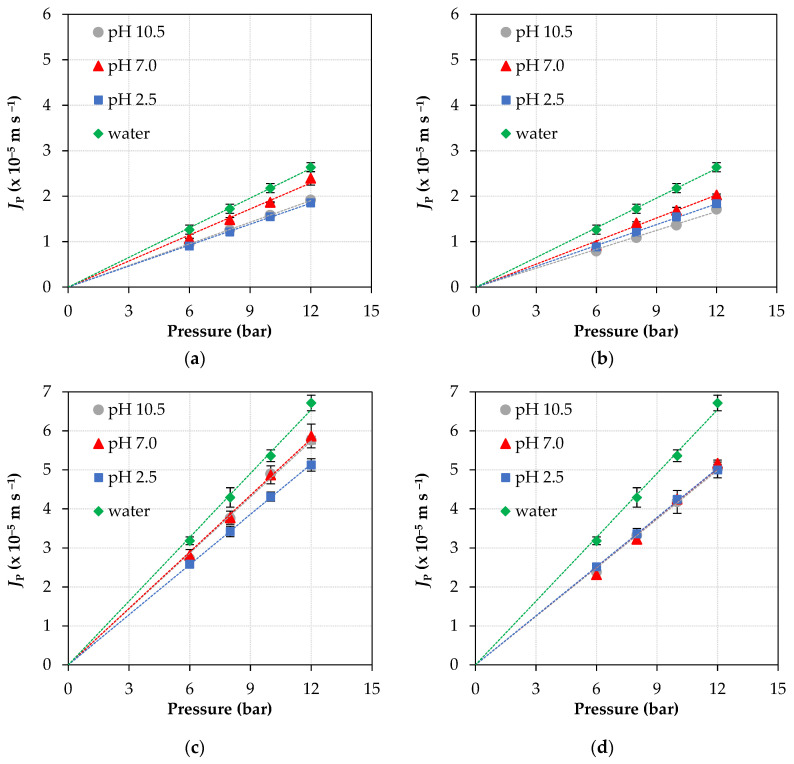
Permeate fluxes as a function of pressure, at three pH conditions, with NF90 (top) and NF270 (bottom) membranes: (**a**), (**c**) feed solution with 8 mg L^−1^ ATN; (**b**), (**d**) feed solution with 16 mg L^−1^ ATN.

Figure 2 shows the variation of permeate fluxes as a function of the operating pressure, for pure water and ATN solutions of 8 and 16 mg L^−1^ at three pH conditions (2.5, 7.0, and 10.5), for NF90 and NF270 membranes. The permeate fluxes for both membranes and both ATN solutions vary linearly with operating pressure for all evaluated pH conditions. In addition, the corresponding straight-line slopes increase with increasing feed concentration, are slightly below that with pure water, and are dependent on the solution pH. 

As reported in earlier studies [24,35], NF90 and NF270 are thin-film polyamide membranes and have amphoteric characteristics, with an isoelectric point at approximately pH 3.5–5.0. Therefore, for pH values below the isoelectric point, the membranes are positively charged due to the protonation of amine groups, while for pH above the isoelectric point, the membranes assume a negative charge due to the deprotonation of carboxyl groups and the zeta potential becomes more negative as the pH arises. This amphoteric characteristic of membranes plays an important role in nanofiltration, promoting electrostatic interactions with ionizable solutes such as Atenolol, for example, which can result in completely different performances—either in terms of productivity (permeate flux, displayed in Figure 2) or selectivity (rejection of contaminants)—depending on the pH range of the aqueous matrix (water or wastewater) that is being treated.

Indeed, studies have shown that organic solutes (such as ATN) can strongly interact with the active layer of polyamide NF/RO membranes [36,37] and these interactions can vary with pH [8,38], influencing both permeate flux and rejection of certain contaminants. More important, the affinity of the solute for the membrane can be evaluated by determining the *B* parameter, so that the higher the value of *B*, the greater the affinity [25]. 

**Figure 3:**
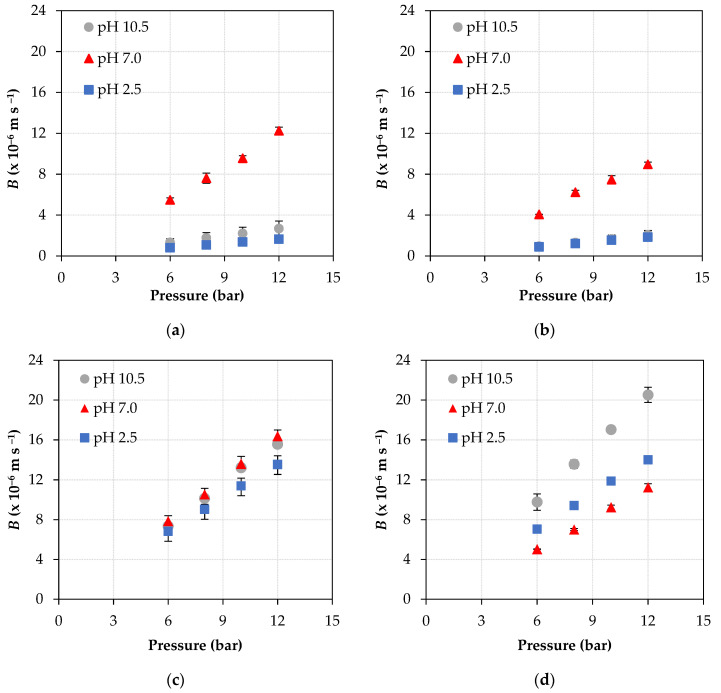
Variation of the *B* parameter as a function of pressure, at three pH conditions, with NF90 (top) and NF270 (bottom) membranes: (**a**), (**c**) feed solution containing 8 mg L^−1^ ATN; (**b**), (**d**) feed solution containing 16 mg L^−1^ ATN.

Within this context, Figure 3 shows the values of *B* parameter obtained at the different pH conditions (2.5, 7.0 and 10.5), operating pressures (6, 8, 10, and 12 bar), and ATN concentrations (8 and 16 mg L^–1^). It is observed that at pH 7.0, the NF90 membrane, at both concentrations and pressures evaluated, presents the highest values of the *B* parameter. That is, at neutral pH and at all pressures evaluated, a greater affinity between ATN and NF90 membrane than at pH 2.5 and pH 10.5 is observed. This higher affinity can be explained by means of electrostatic interactions, since the membrane has an isoelectric point, and the pharmaceutical compound under study, depending on the pH, is in its cationic or neutral form.

It is known that at a pH below the isoelectric point, this membrane assumes a positive charge, and that at a pH above the isoelectric point, it assumes a negative charge [24]. It is also known that the p*K*a of ATN is 9.6 (Table 1), that is, a pH at which 50% of ATN is in its cationic form and the other 50% in its neutral form. Therefore, at pH 2.5, all the ATN is in its cationic form and the membrane is positively charged, thus causing a repulsion between the solute and the membrane, consequently resulting in a lower *B* value. Likewise, at pH 10.5, most of the ATN (about 90% [23]) is in its neutral form and the membrane is negatively charged, with the results of *B* showing that there is a low solute/membrane interaction at this pH. On the other hand, at pH 7.0 the membrane is negatively charged and the ATN is completely in its cationic form [23], resulting in an attraction between the membrane and the solute, which is evident with the high *B* values obtained.

However, the same does not occur with the NF270 membrane. The *B* values are very close in all pH evaluated for the lowest feed concentration, 8 mg L^−1^ ATN, with a small decrease on it at pH 2.5. At this pH, the highest rejections occurred and both ATN and the membrane surface layer are positively charged, so there will be a repulsion between the solute and the membrane resulting in lower *B* values. Furthermore, higher permeate fluxes were obtained at pH 7.0 and 10.5 than at pH 2.5. In addition, very similar ATN rejections at pH 7.0 and 10.5 were observed, which resulted in slightly higher *B* values at pH neutral-basic, since this parameter depends on both permeate flux and intrinsic rejection, as represented in Equation (9). Importantly, for all conditions evaluated and for both membranes, intrinsic and apparent rejections were very close.

On the other hand, for the NF270 membrane, at the highest feed concentration (16 mg L^−1^ ATN) and pH 10.5, the *B* values are higher than those obtained at the other pH (2.5 and 7.0). This difference, which also occurs in relation to the NF90 membrane, can be explained by the fact that the NF270 membrane, at basic pH, suffers much more from electrostatic repulsion in the membrane matrix, causing an increase in its pore size and consequently a lower rejection of the neutral solute and also a higher solute/membrane interaction, resulting in higher *B* values [39]. Moreover, for the NF270 membrane, at the highest feed concentration, 16 mg L^−1^ ATN, the lowest *B* values were obtained at pH 7.0, unlike what occurs in the NF90 membrane. Remembering that *B* depends on permeate flux and intrinsic rejection, at pH 7.0 and pH 2.5, the NF270 membrane showed very similar rejections, around 80%; however, the permeate fluxes were slightly lower at pH 2.5 than at pH 7.0, thus reflecting lower *B* values at pH 2.5 than at pH 7.0.

It is important to highlight that for both membranes and conditions evaluated, the *B* parameter displayed a linear dependence on pressure; that is, the higher the pressure applied, the higher the value of *B*. This behavior is due to the fact that with the increase in pressure, the permeate flux also increases [23] and, according to Equation (9), *B* is proportional to the permeate flux. Furthermore, it has also been reported in the literature that conditions of high permeate fluxes can result in a higher incidence of concentration polarization [17,33,40]; that is, a higher concentration of solute at the boundary layer adjacent to the membrane, resulting in higher membrane/solute interactions.

**Figure 4:**
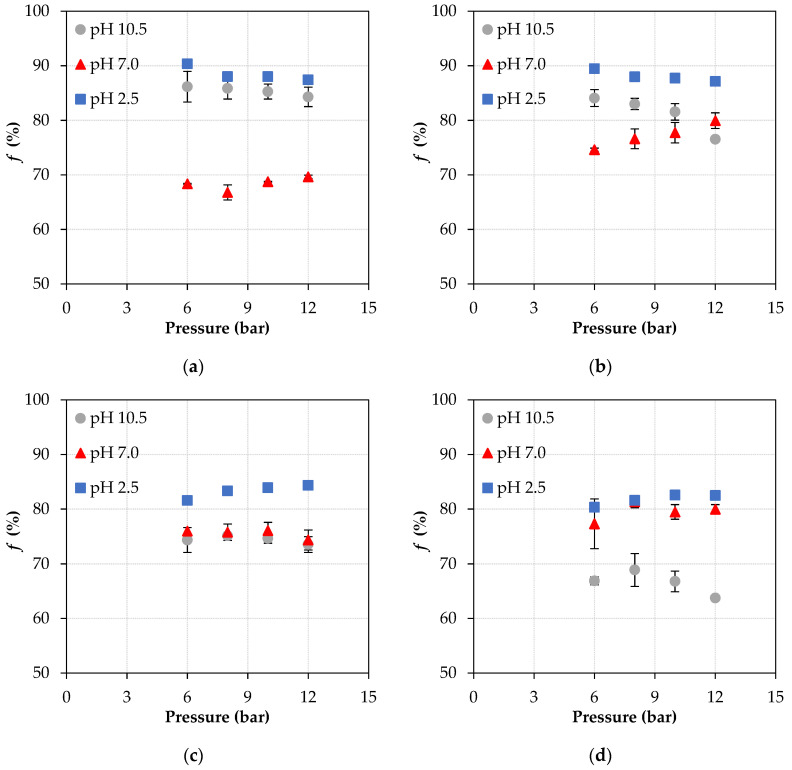
Apparent rejection coefficient of ATN as a function of pressure, at three pH conditions, with NF90 (top) and NF270 (bottom) membranes: (**a**), (**c**) feed solution with 8 mg L^−1^ ATN; (**b**), (**d**) feed solution with 16 mg L^−1^ ATN.

Figure 4 shows the behavior of ATN rejection in relation to operating pressure, solution pH, and two ATN concentrations (8 and 16 mg L^−1^), for NF90 and NF270 membranes. 

It is known that in NF90, identified as a tight NF membrane, steric hindrance plays an important role in rejection, considering that this membrane has a smaller pore size (around 0.34 nm), when compared to other NF membranes [8,24]. Nonetheless, electrostatic interactions also play an important role in the selectivity of this membrane. As can be seen in Figure 4, the best rejection results occur at pH 2.5 and 10.5. At pH 2.5, both the membrane and Atenolol are positively charged, so there is a repulsion between them and, consequently, a greater rejection is reached. At pH 10.5, however, a large fraction of ATN is in its neutral form, while the membrane is negatively charged, with no repulsion or attraction. On the other hand, at pH 7.0, the lowest rejection values are observed, which can be attributed to electrostatic interactions, since at this pH the membrane is negatively charged and ATN is in its cationic form (positively charged). As a consequence, there will be an attraction between them, which results in a greater amount of ATN near the membrane, facilitating its passage through the membrane and, consequently, obtaining lower rejection values.

Similarly, although NF270 is known as a loose membrane and has a larger pore size (about 0.42 nm) and 400 Da MWCO [8,24], it also had the best values of rejection, at both ATN concentrations studied, at pH 2.5. At this pH, both the membrane and the pharmaceutical compound under study are positively charged, causing repulsion and consequently high rejection values. However, unlike what happens with NF90, at pH 10.5 and at the concentration of 16 mg L^–1^ of ATN, NF270 has the lowest rejection values (below 70%). In this scenario, in addition to NF270 being a membrane with a larger pore size than NF90, as mentioned above, NF270 at basic pH conditions suffers much more from the repulsion in the membrane polymer matrix, resulting in an increase in its pore size and consequently lower values of rejection of the neutral compound [39]. In a previous study [17], we observed that the concentration polarization also interferes with the rejection coefficient; that is, under conditions of higher concentration polarization, the solute flux across the membrane is greater and, consequently, the rejection of ATN is lower.

Summing up, the apparent rejection results (Figure 4) are inwardly related to the *B* parameter results (Figure 3) and are dependent on pH and operating pressure. In general, under the conditions in which the highest values of *B* were observed, the lowest ATN rejection values were achieved, and this behavior was governed by electrostatic solute/membrane interactions.

## 5. Conclusions

There are several factors influencing the rejection coefficient in nanofiltration, including size exclusion and electrostatic and hydrophobic interactions, which are dependent on the solute, membrane, and aqueous matrix properties. Therefore, the present study was focused on the evaluation of the influence of pH on the removal of the Atenolol as a model of pharmaceutical compound by nanofiltration, as well as its influence on the affinity of the solute for the membrane through the determination of the *B* parameter. Both membranes tested showed the best rejection values at pH 2.5, demonstrating that, in addition to steric hindrance, electrostatic interactions play a very important role in the nanofiltration process. In addition, through the *B* parameter, it was possible to verify the solute/membrane affinity at the different pH conditions and membranes evaluated, showing that at pH 2.5 the solute/membrane affinity is lower and, consequently, the rejection coefficient is higher. Therefore, the importance of determining this parameter is highlighted, along with the other operational parameters and hydrodynamic conditions, in order to understand how the nanofiltration process takes place, the influence of the various factors involved in it, and above all, to optimize this process to get the best cost-benefit.

## Figures and Tables

**Table 1:** Main characteristics of Atenolol (ATN).

Characteristic	Description
Molecular formula ^1^	C_14_H_22_N_2_O_3_
Structural formula ^1^	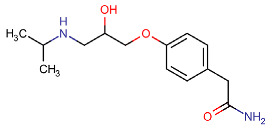
Molecular weight (Da) ^1^	266
Stokes radius—r_s_ (nm) ^2^	0.34
Water solubility at 25 °C (g L^−1^) ^3^	13.5
Log *K*_OW_^1^	0.16
p*K*a ^1,2^	9.6

^1^ From [31]; ^2^ From [23]; ^3^ From [32].

## Data Availability

The data that support the findings of this study are available from the corresponding author upon reasonable request.
